# Chicago Pathological Society

**Published:** 1883-09

**Authors:** J. H. Tebbetts

**Affiliations:** Secretary


The society, on motion, adjourned.

J. H. TEBBETTS, *Secretary.*

---

### ARTICLE X.

### CHICAGO PATHOLOGICAL SOCIETY.

July 9. Regular meeting. The meeting was called to order by the President, Dr. Angear. The minutes were read and approved.

The following named gentlemen were elected to membership:

Drs. R. N. Hall, J. E. Harper, E. E. Holroyd and W. A. Synon.

In the unavoidable absence of Dr. E. P. Murdock, who was to have given the society a verbal report of an interesting case of infantile tetanus, Dr. Angear in a few words related what he knew of the case, having been called in consultation one hour before death occurred. The case occurred in a well-to-do family. A normal labor, without complications, was followed by no bad symptoms in the child till the sixth day, death supervening the evening of the seventh. The slightest current of air sufficed to throw the child into a most intense convulsion, extreme opisthotonos. The only lesion, of course, must have been the condition of the navel, and there was a peculiar redness around the umbilicus of an irregular shape.

The disease is coincident and probably excited by filth and foul air.

In London, over one hundred years ago, one out of six children died in the maternity hospitals within the first ten days with this peculiar disease.

About the same proportion of cases presented at Dublin at the same time. On sanitary improvements in these hospitals being introduced, the death rate fell from 2,000 to 500.

The Society then listened to the reading of a paper by Dr. Robinson, entitled " Myxœdema."

This rare and peculiar disease first began to be studied and differentiated in 1833 by Gall, but was then supposed to be a form of cretinism. In this country but five cases have been reported. Dr. Robinson then gave a detailed report of two interesting cases.

CASE I. Female, age 40; had several children; general health fair; face greatly and uniformly swollen with an eruption similar to that of arsenical poisoning; urine scanty, no albumen, no casts; speech slow, retarded; mental processes slow; no loss, but an impairment of coördination.

CASE II. Female, age 52, married; had several children, good general history; for eleven years has got easily frightened; mentally depressed by trifles, but not melancholic; rather lethargic, urticaria appears when stomach is disordered; throat dry, eyes swell, then face, then whole body simultaneously; perspiration absent, skin partially numb; taste impaired, also hearing and vision; menopause over; gait unsteady, but no ataxic symptoms; features have lost their play from the œdema, pupils normal; speech slow and hesitating; mental action slow; cheeks blush continually, eyelids pale and waxy.

The author then enumerated among the points of diagnosis that the cases reported have all been adult females; that the appearance of the face is like that in renal disease; the cheeks are usually flushed; the œdema is not that of dropsy—it appears simultaneously all over the body. The intellect becomes sluggish at an early stage; the voice monotonous; coördination tardy, but not lost.

Death occurs from coma uræmia, or inanition. The etiology is at present doubtful; perhaps a neurosis of the motor system, together with disorder of the lymphatic system; according to some, an unrecognized albuminuria; or an accumulation of mucin in the connective tissue, deadening the action of the nerves.

*Post-mortem* changes do not reveal the cause of the disease. Degeneration of the anterior columns of the cord has been found. The arterial coats thickened; the stroma of the liver, spleen and kidney increased. The prognosis would be unfavorable. Renal affections are usually present in later stages. Little can be done way of treatment; he would advise milk diet, tonics and hygienic surroundings. Nitro-glycerine has been used to reduce capillary caliber.

Dr. Bennett remarked his interest in the reading of the paper, and inquired whether Bright's Disease could not co-exist, and was answered by Dr. Robinson that it could, and did, toward the close of life. The œdema does not pit on pressure nor extend by gravitation, as in Bright's disease.

Dr. Bennett then moved that the author be voted the thanks of the society and a copy of the papers furnished for publication. Carried.

Dr. Bennett then reported two interesting hospital cases, one of lumbago, severe, cured in two days by salicylate of soda, 30 gr. every two hours.

The second case, one of syphilis, which ran the whole course from initial lesion to secondary symptoms, iritis, meningitis, and death in six months.

Among the members present were Drs. Angear, Bennett, Brophy, Dobbin, Harper, Sanders, Robinson, Tebbetts, and several visitors. The society, on motion, adjourned.

J. H. TEBBETTS, *Secretary.*

---

A HOMŒOPATHIC physician at Vienna has left a large sum of money for the establishment of a homœopathic chair in the Vienna University, but the Austrian Government has declined to accept the money.

